# A systematic review of the latent structure of the Center for Epidemiologic Studies Depression Scale (CES-D) amongst adolescents

**DOI:** 10.1186/s12888-021-03206-1

**Published:** 2021-04-19

**Authors:** Joanna M. Blodgett, Chantelle C. Lachance, Brendon Stubbs, Melissa Co, Yu-Tzu Wu, Matthew Prina, Vivian W. L. Tsang, Theodore D. Cosco

**Affiliations:** 1 MRC Unit for Lifelong Health and Ageing at UCL, London, UK; 2 Independent Researcher, Toronto, Canada; 3 King’s College London, Health Service and Population Research Department, Institute of Psychiatry, Psychology & Neuroscience, London, UK; 4 Physiotherapy Department, South London and Maudsley NHS Foundation Trust, London, UK; 5 Faculty of Medicine, University of British Columbia, Vancouver, Canada; 6 Oxford Institute of Population Ageing, University of Oxford, Oxford, UK; 7 Gerontology Research Centre, Simon Fraser University, Vancouver, Canada

**Keywords:** Adolescents, Center for Epidemiologic Studies-Depression Scale, Depression, Factor structure, Psychometric

## Abstract

**Background:**

The Centre for Epidemiologic Studies Depression Scale (CES-D) is a commonly used psychometric scale of depression. A four-factor structure (depressed affect, positive affect, somatic symptoms, and interpersonal difficulties) was initially identified in an American sample aged 18 to 65. Despite emerging evidence, a latent structure has not been established in adolescents. This review aimed to investigate the factor structure of the CES-D in adolescents.

**Methods:**

We searched Web of Science, PsychINFO and Scopus and included peer-reviewed, original studies assessing the factor structure of the 20-item CES-D in adolescents aged ≤18. Two independent researchers screened results and extracted data.

**Results:**

Thirteen studies met the inclusion criteria and were primarily from school-based samples in the USA or Asia. Studies that conducted confirmatory factor analysis (CFA; *n* = 9) reported a four-factor structure consistent with the original factor structure; these studies were primarily USA-based. Conversely, studies that conducted exploratory factor analysis (EFA) reported distinct two or three factor structures (*n* = 4) and were primarily based in Asia.

**Limitations:**

Studies in a non-English language and those that included individuals aged > 18 years were excluded. Ethnic or cultural differences as well as different analytical methods impacted generalisability of results. The use of CFA as the primary analysis may have biased towards a four-factor structure.

**Conclusions:**

A four-factor CES-D structure was an appropriate fit for adolescents in Western countries; further research is required to determine the fit in in Asian countries. This has important implications for clinical use of the scale. Future research should consider how cultural differences shape the experience of depression in adolescents.

**Supplementary Information:**

The online version contains supplementary material available at 10.1186/s12888-021-03206-1.

## Background

Depression is a common mental health problem world-wide, with a projected global prevalence of 4.4% [1]. The World Health Organisation estimates that major depression will be the leading cause of disease burden by 2030 [2, 3]. Rates of underdiagnosed and undertreated depression are higher in adolescents than in adults [4], which is concerning given the link between adolescent depression and increased risk of suicide, lower educational attainment, and higher likelihood of smoking, obesity, and drug or alcohol misuse [4–7]. In addition to the immediate consequences of adolescent depression (e.g. substance abuse, suicide), there are substantial, and longer term, negative impacts on adult mental and physical health [7].

In order to measure depression in adolescents, feasible and accurate assessment of depressive symptoms is necessary. The Centre for Epidemiologic Studies Depression Scale (CES-D) is a self-reported, psychometric scale intended to identify the frequency and severity of depressive symptoms [8]. Consisting of 20 items measured on a four-point Likert scale, this measure has been used across age group, country and in both community and institutionalised samples. Originally developed by Radloff [8], the original factor structure included four factors: depressed affect (7 items; e.g. feeling lonely or sad, crying spells), positive affect (4 items; e.g. feeling hopeful or happy), somatic complaints (7 items; e.g. decreased appetite, restless sleep or difficulty getting going) and interpersonal difficulties (2 items; e.g. feeling that others were unfriendly or feeling disliked by others).

The CES-D factor structure was initially established in a sample of community dwelling, American adults, aged 18+ and there is no consensus on its validation in a younger sample. Depression may manifest differently in younger individuals compared to the general adult population [9, 10]. As such, it is critical to test the factorial construct validity of the CES-D scale. By identifying the factor structure of the CES-D in adolescents, one can establish whether the same four-factor structure is applicable and, consequently, determine if this scale is suitable for use in a younger population. We aimed to systematically review and summarise all existing literature that analyses the latent structure of the CES-D in adolescents (≤18 years). We hypothesised that the CES-D factor structure in adolescents will be consistent with the original four-factor structure posited by Radloff [8].

## Methods

Scopus, and PsycInfo (via the Ovid platform) databases alongside the Web of Science collection of databases were used to search for all original research articles that reported data on the factor structure of the 20-item CES-D scale. For each database, default search fields were used. Scopus used article title, abstract and keywords; PsycInfo used keywords; Web of Science databases used Topic, which includes abstract, author keywords, and Keywords Plus. The search spanned all studies published from inception to July 2019. Using Boolean operators, the search strategy consisted of the following terms: (“CES-D” OR CES-D OR “Center for Epidemiologic Studies Depression Scale”) AND (validation OR psychometric OR factor structure OR latent structure OR dimension*).

To be included in the review, studies had to meet the following inclusion criteria: 1) peer-reviewed, 2) original analysis of the factor structure, 3) full 20-item CES-D, and 4) sample aged ≤18 years. Studies were excluded if they were published in a non-English language or did not meet the inclusion criteria (e.g. 10-item scale, a sample aged 15–20). Non-English studies were excluded due to resource limitations, although studies that utilised non-English versions of the CES-D scale were still included. Screening was independently conducted by two authors in two phases; first, a title-abstract screening followed by a full text screening. Demographic characteristics (e.g. age, sex, country, setting), latent structure characteristics (e.g. method of identifying latent structure, number of factors, items in each factor) and other psychometric features (e.g. Cronbach’s alpha) were also independently extracted by two authors. Any discrepancies were resolved by a third author. Risk of bias of the outcome was not assessed due to the nature of the psychometric scale assessment.

## Results

**Fig. 1:**
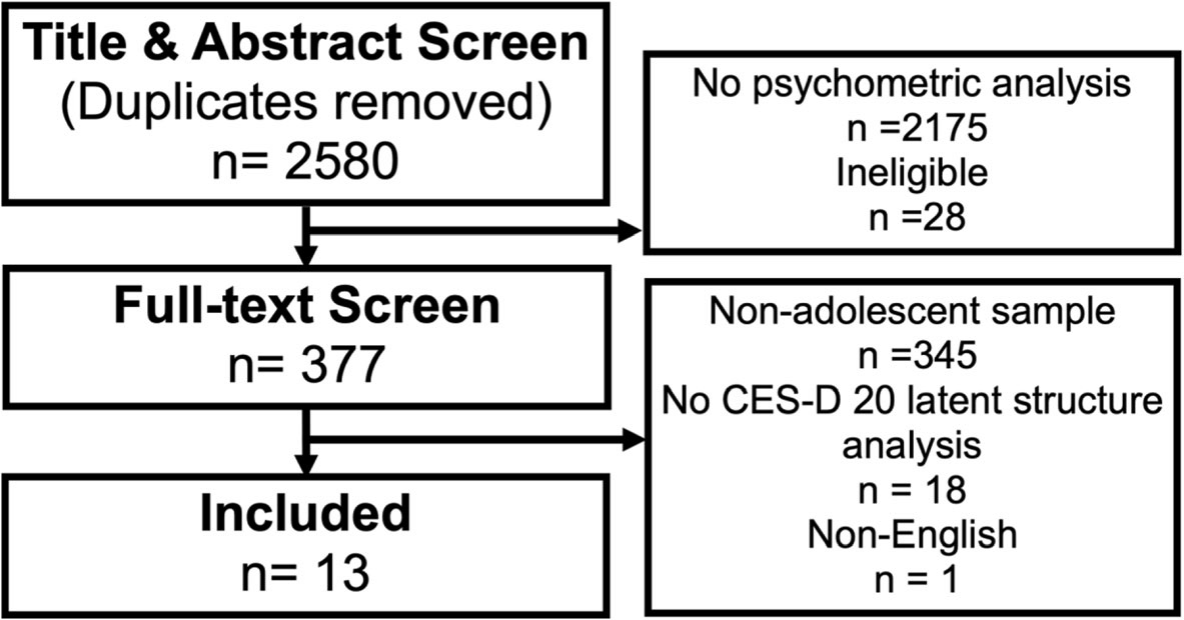
Article inclusion flowchart

After removal of duplicates (using the “Find duplicates” function and hand searching in EndNote), a total of 2580 studies were identified in the search process. Of these, 377 underwent a full text screening and 13 met the full inclusion criteria (Fig. 1). All demographic characteristics of the studies are provided in Table 1. The average sample size was 3292 (range: 148–10,691) and the mean age ranged from 12.2 to 16.2; note that not all studies reported the mean age. The proportion of males and females was similar (mean: 47.5% female), with most studies having an equal distribution. Most studies were based in the USA (*n* = 6) or Asia (*n* = 5; Taiwan, Jordan, Malaysia, Korea, China), with one study in Germany and one cross-continental study in Turkey. Consequently, more than half of the studies utilised a non-English CES-D that was translated from the original. Seven of the thirteen studies used data collected in schools [14, 16–18, 21–23], while one drew participants from a nationally representative study [12] and the remaining five utilised secondary data from nationally or locally representative studies [11–13, 19, 20]. Two studies reported that there were no physical morbidities or characteristics of the sample that could influence the results [11, 13], while the remaining studies did not provide any details on this.

**Table 1:** Study characteristics of included studies (*n* = 13)

Author	N	Age (Mean ± SD; Range)	% female	Country	Study sample and/or method of data collection	Language of scale
Barkmann et al. (2008) [11]	2863	Mean: NR Range: 7–17	–	Germany	German Health Interview and Examination Survey for Children and Adolescents (random sampling)	German
Cheng et al. (2012) [12] (Cheng et al., 2012)	10,116	Mean: NR Range: 12–18	51.1%	Taiwan	Project for Health of Adolescents in Southern Taiwan (random sampling)	Chinese
Crockett et al. (2005) [13]	10,691	Mean: NR Range: 12–18	50.8%	USA	National Longitudinal Study of Adolescent Health (subsample of Anglo-, Mexican-, Cuban- and Puerto Rican- Americans)	English
Dardas et al. (2019) [14]	3292	Mean: 15.7 ± 1.1 Range: 13–17	53.6%	Jordan	Questionnaires administered in schools (type = NR)	Arabic
Faulstich et al. (1986) [15]	148	Mean: 13.9 ± 2.3 Range: 8–17	22.0%	USA	Child and adolescent psychiatric inpatients in large southern US city	English
Ghazali et al. (2016) [16]	931	Mean: 15 ± 1.5 Range: 13–17	62.2%	Malaysia	Questionnaires administered in schools (type = NR)	Malaysian
Heo et al. (2018) [17]	1884	Mean: 14.8 ± NR Range: 13–16	33.5%	Republic of Korea	Questionnaires administered to middle schools	Korean
Li et al. (2010) [18]	313	Mean: 9.9 ± NR Range: 8–12	48.6%	China	Questionnaires administered to primary schools	Chinese
Motl et al. (2005) [19]	2416	Mean: 12.7 ± 0.4 Range: 10–14	51.8%	USA	TEENS Study (school-based, group randomized trial)	English
Phillips et al. (2006) [20]	3709	Mean: 12.9 ± NR Range: 10–14	50.5%	USA	TEENS Study (school-based, group randomized trial)	English
Roberts et al. (1990) [21]	2160	Mean: 16.2 ± NR Range: 14–18	55.4%	USA	Questionnaires administered to public and parochial high schools	English
Skriner and Chu (2014) [22]	881	Mean: 12.5 ± 0.4 Range: 11–14	45.0%	USA	Questionnaires administered to suburban and urban middle schools	English
Tatar et al. (2013) [23]	583	Mean: 12.2 ± 1.9 Range: 8–15	49.1%	Turkey	Questionnaires administered to primary schools	Turkish

*SD* standard deviation, *NR* not reported in study, *TEENS* Teens Eating for Energy and Nutrition at School, *USA* United States of America

Table 2 describes the analyses used as well as details on the loadings and structure of factors. Seven studies primarily conducted confirmatory factor analyses (CFA, i.e., hypothesis-driven) while one study performed exploratory factor analyses (EFA; i.e. data-driven), one study used principal component analysis (PCA) and four studies used both CFA and EFA. As their names suggest, CFA assesses an a priori selected structure, while EFA has no prior factor structure [24]. Where some studies identified or tested multiple factor structures, the model that was the best fit reported. Further detail on analytical software, rotation method (EFA) and goodness of fit indices are provided in Supplementary Table 1. LISREL and SPSS were the most common software used, while chi square and comparative fit index (CFI) were the most commonly reported goodness of fit indices.

**Table 2:** Method of analyses, loading and structure of factors for included studies (*n* = 13)

Author	Method	Cronbach’s alpha	Number of factors	FACTORS				
				Depressed affect	Positive affect	Somatic	Interpersonal	Did not load
*Original paper:* Radloff (1977) [8]	PCFA		4	3,6,9,10,14,17,18	4,8,12,16	1,2,5,7,11,13,20	15,19	–
Barkmann et al. (2008) [11]	CFA	0.67	4	3,6,9,10,14,17,18	4,8,12,16	1,2,5,7,11,13,20	15,19	–
Li et al. (2010) [18]	CFA	Test: 0.82 Retest: 0.85	4	3,6,9,10,14,17,18	4,8,12,16	1,2,5,7,11,13,20	15,19	–
Motl et al. (2005) [19]	CFA	NR	4	3,6,9,10,14,17,18	4,8,12,16	1,2,5,7,11,13,20	15,19	–
Phillips et al. (2006) [20]	CFA	NR	4	3,6,9,10,14,17,18	4,8,12,16	1,2,5,7,11,13,20	15,19	–
Roberts et al. (1990) [21]	CFA	Male: 0.88 Females: 0.91	4	3,6,9,10,14,17,18	4,8,12,16	1,2,5,7,11,13,20	15, 19	–
Cheng et al. (2012) [12]	CFA	NR	4	3,6,9,10,14,17,18	4,8,12,16	1,2,5,7,11,13,20	15,19	–
Crockett et al. (2005) [13]	CFA, EFA	NR	4	3,6,9,10,14,17,18	4,8,12,16	1,2,5,7,11,13,20	15,19	–
Skriner et al. (2014) [22]	CFA	0.83	4	3,6,9,10,14,17,18	4,8,12,16	1,2,5,11,13,20	15,19	7
Tatar et al. (2013) [23]	CFA, EFA	0.74	4	3,6,9,10,14,17,18	4,8,12,16	1,2,5,11,13,20	15,19	7
Faulstich et al. (2016) [15]	PCFA	Age < 13: 0.77 Age 13+: 0.86	3	1,6,9,10,14,17,18	4,8,12,16	2,5,7,11,15,19,20	–	3, 13
Ghazali et al. (2016) [16]	EFA	0.85	3	9, 10,13,14,15,17,18,19,20	4,8,12,16	1,2,3,5,6,7,11	–	–
Heo et al. (2018) [17]	EFA, CFA	0.88	3	3,6,10,11,13,14,15,17,18,19	4,8,12,16	1,2,5,7,9,20	–	–
Dardas et al. (2019) [14]	EFA, CFA	0.88	2	1, 2, 3, 5, 6, 9, 10, 11, 14, 15, 17, 18, 19, 20	4,8,12,16	–	–	7, 13

*NR* not reported in study, *PCFA* principal component factor analysis, *CFA* confirmatory factor analysis, *EFA* exploratory factor analysis

Nine studies provided evidence that Radloff’s four-factor structure was an appropriate fit for the data. Of these nine studies, seven reported an identical structure [11–13, 18–21] while two studies found that item 7 “I felt that everything I did was an effort” did not load onto any factor [22, 23]. Three studies reported unique three-factor structures, each consisting of a depressive affect, positive affect and somatic symptoms factor [15–17], and one study reported a two-factor structure of depressive and positive affects.

## Discussion

The results of this systematic review provide supportive evidence on the validity of the original four-factor structure of the CES-D in adolescent populations. Nine of the 13 studies were consistent with Radloff’s original four-factor structure. An additional three studies proposed distinct three-factor structures [14–17] while one study proposed a two-factor structure (Dardas et al., [14]). All 13 studies reported an identical positive affect factor (items 4, 8, 12, 16) but demonstrated differences across other factors. Cultural and ethnic differences may underlie these major differences in factor structure; this is discussed in further detail below and has important implications when considering global use of the CES-D. Use of the CES-D in adolescents appears to be most appropriate when used in community-dwelling individuals in Western countries.

### Studies reporting a four-factor structure

All nine studies that provided evidence that Radloff’s four-factor structure was an appropriate fit for the data used CFA as their initial approach. Studies that proposed an alternative structure performed EFA or PCFA as their primary analyses. Six studies exclusively tested Radloff’s four-factor structure using CFA and did not consider other factor structures [11, 18, 19, 21, 22]. The studies that did compare Radloff’s structure to alternative options suggested that the four-factor structure remained the best fit, although there was adequate model fit in several other two and three- factor structures [12, 20]. These studies were primarily based in an American sample (*n* = 5), with one study from each of Germany, Turkey, Taiwan and China.

### Studies reporting a two or three-factor structure

Four studies proposed distinct two or three-factor structures. In contrast to studies above, each sample was ethnically diverse, drawn either from schools in the Republic of Korea, Malaysia or Jordan or from an in-patient psychiatric facility in USA. How these ethnic or cultural differences may underlie major differences is explored in further detail below.

Heo et al. [17] and Ghazali et al. [16] used EFA to identify distinct three-factor structures as the optimal factor structure for the CES-D in school-based samples in the Republic of Korea and Malaysia, respectively. Heo and colleagues [17] subsequently used CFA to compare the three-factor structure to several other structures that had been suggested in the literature, including Radloff’s four-factor structure. There was no evidence that one structure was a better fit than another, suggesting that multiple factor structures may be valid for use in adolescents. Conversely, Ghazali et al. [16] did not assess the fit of other factor structures; thus, did not determine if the four-factor structure would have been an acceptable fit. Although there are some similarities in factor loadings between these three-factor structures, there remain distinct differences in items that could be attributable to cultural differences between these two Asian countries.

Faulstich et al. [15] applied a principal component analysis (PCA) approach to identify three distinct factors: a happiness dimension (identical to the positive affect factor in Radloff’s proposed structure), behavioural component of depression (similar to depressive affect) and a cognitive component of depression (combined somatic and interpersonal). However, the generalisability of this factor structure to the general adolescent population is limited for several reasons. First, the sample was drawn from two psychiatric inpatient facilities in the Southern USA and consequently is heterogeneous both in age (8 to 17 years old) and in diagnoses, which included conduct disorder, major depression, schizophrenia and atypical psychosis amongst others. This may explain why interpersonal items (i.e. item 15: people were unfriendly and item 19: “people disliked me”) loaded on to the somatic factor and why items 3 (i.e. “could not shake off the blue”) and 13 (i.e. “talked less than usual)” failed to load on to any factor. Furthermore, the authors utilised a version of the CES-D that was translated to be more comprehensible for children and adolescence. For example, “I felt that I could not shake off the blues even with help from my family or friends” in the original CES-D was translated to “I wasn’t able to feel happy, even when my family or friends tried to help me feel better.” This could have changed how individuals responded to each item. Finally, PCA assumes that the total variance is equal to the common variance, whereas EFA and CFA consider both common variance and unique variance. Differences in both the operationalisation of the questionnaire and the subsequent analyses may limit generalisability.

Dardas et al. [14] was the only study to propose a two-factor structure of positive and negative affect. The sample of Arab adolescents failed to distinguish between depressed affect symptoms, somatic symptoms and interpersonal difficulties of positive and negative affect items. Cultural differences such as decreased expression of emotions or inability to recognise both cognitive and physical symptoms of depression may underlie these differences [25].

### Ethnic or cultural differences

The review summarises emerging evidence that the CES-D is not psychometrically equivalent across adolescent cultural and ethnic groups. These cross-cultural findings are consistent with those seen in reviews of CES-D in older adults [26]. Positive effect was the only domain consistently seen across all cultures. Evidence suggests that depression may be driven by psychological aspects in Western cultures (i.e. USA) and by somatic factors in Eastern cultures (i.e. China, Republic of Korea) [27]. Evidence suggests that Western cultures can better distinguish between psychological, somatic and interpersonal symptoms of depression, compared to Asian or Arab samples [25, 28–30]. Individuals from Western cultures are thought to overemphasise the distinction between depressive symptoms of the mind and body, with a particular emphasis on the affective or psychological aspects [30].

Even within a single country, there can be considerably diversity in depressive symptoms. For example, Crockett et al. [13] examined how the structure may change amongst different American ethnic groups and found that the four-factor structure fit in Anglo and Mexican Americans, whilst a separate four-factor structure demonstrated the best fit in Cuban Americans and a five-factor structure was optimal in Puerto Rican Americans. Originally developed in an adult Caucasian sample, Radloff’s four-factor structure appears to be appropriate for an ethnically similar adolescent sample but further investigation of structural invariance across culture is necessary to support its translation and continual use in different cultures.

### Limitations

Several limitations must be considered in the interpretation of these results with respect to the manner in which the search was conducted and the findings in the studies captured in the review. We searched the Scopus database, which includes MEDLINE; however, Scopus does not offer the same subject heading search feature offered by MEDLINE and suggested by the Cochrane Handbook. Although best efforts were made to be comprehensive in the search strategy, articles may not have been captured by the search due to the keywords used in the search strategy and/or the absence of subject heading searches. Additionally, the total number of items captured in the search strategy was not reported, only the number of articles remaining after duplicates were removed.

First, studies that were published in a non-English language or that captured adolescents in a wider age range (e.g. aged 13–20) were not included; this may have resulted in study selection bias. Next, there was substantial heterogeneity of sample characteristics- most notably, country, age, and language of the CES-D. These differences could have induced differences in factor structure, limiting comparability between studies. While different fit indices and software appeared to have no effect on the final results, inference is limited due to differing reports of goodness of fit indices. Finally, there was a bias towards a four-factor structure in studies who utilised a hypothesis-driven approach (i.e. CFA). While this approach suggested that a four-factor structure was appropriate, a data-driven approach (i.e. EFA) may have identified more optimal factor structures.

## Conclusions

This systematic review summarised the evidence from 13 studies on the factor structures of the CES-D in adolescents aged 18 years or younger. Most of the evidence provided support for the four-factor structure. Ethnicity and culture had a significant impact on factor structure, with clear evidence that Western and non-Western countries experienced depressive, somatic, and interpersonal symptoms differently. To the authors’ knowledge, this review captured all English studies examining factor structure of the full 20 item CES-D in youth aged 18 years and younger. A recent systematic review demonstrated that the factor structure of the CES-D could be applied to adults over the age of 65 [26]. This review shows that a similar factor structure may be utilised in a Western-based sample aged 18 years and under, although there are clear cultural and ethnic differences. Future research should consider how these differences shape the experience of depression to ensure that the CES-D is adequately capturing depression across culture.

## Supplementary Information

**Additional file 1.**

## Data Availability

Data sharing is not applicable to this article as no datasets were generated or analysed during the current study.

## Abbreviations

CES-D: Center for Epidemiologic Studies-Depression Scale
CFA: confirmatory factor analysis
EFA: exploratory factor analysis
PCA: principal component analysis
