# Supplementary material for: A systematic review of the latent structure of the Center for Epidemiologic Studies Depression Scale (CES-D) amongst adolescents
Source: BMC Psychiatry. 2021 Apr 19;21:197. doi: 10.1186/s12888-021-03206-1 (PMC8054366; doi:10.1186/s12888-021-03206-1)
Supplement: Supplementary file 1 — Additional file 1. [file 12888_2021_3206_MOESM1_ESM.docx]

| **Supplementary Table 1.** Additional detail on software, rotation method (EFA), and goodness of fit indices for the best fitting model | | | | | | | | |
| --- | --- | --- | --- | --- | --- | --- | --- | --- |
| **Author** | **Rotation method (e.g. varimax, oblimin, promax)** | **Software (name, version)** | **Root mean square error of approximation (RMSEA)** | **Chi-square** | **Tucker-Lewis index (TLI)** | **Comparative fit**  **Index**  **(CFI)** | **Incremental fit**  **index**  **(IFI)** | **Non-normed fit index (NNFI)** |
| Barkmann et al. (2008) | n/a | SPSS 12.0, LISREL 8.54, PRELIS 2.5 | 0.04 | 438.36 | *nr* | 0.99 | *nr* | *nr* |
| Cheng et al. (2012) | n/a | LISREL 8.8 | Girls aged <5: 0.062 | 1619.43 | *nr* | 0.970 | *nr* | 0.969 |
|  |  |  | Girls aged ≥15: 0.067 | 1921.88 |  | 0.970 |  | 0.965 |
|  |  |  | Boys aged <15: 0.067 | 1745.21 |  | 0.970 |  | 0.965 |
|  |  |  | Boys aged ≥15: 0.068 | 1816.02 |  | 0.969 |  | 0.964 |
| Crockett et al. (2005) | *nr* | Mplus (version nr) | 0.096 | 65.56 | *nr* | 0.98 | *nr* | *nr* |
| Dardas et al. (2019) | Varimax | SPSS 21 AMOS 22 | *nr* | *nr* | *nr* | 1.00 | *nr* | *nr* |
| Faulstich et al. (1986) | N/A (examined factorial invariance over time) | | | | | | | |
| Ghazali et al. (2016) | Varimax | SPSS 16.0 | *nr* | *nr* | *nr* | *nr* | *nr* | *nr* |
| Heo et al. (2018) | Varimax | SPSS 22, AMOS 22 | 0.104 | 3577.714 | 0.725 | 0.78 | *nr* | *nr* |
| Li et al. (2010) | n/a | SPSS 16.0 | 0.06 | *nr* | *nr* | 0.96 | *nr* | *nr* |
| Motl et al. (2005) | n/a | LISREL 8.50. | 0.03 | 5705.81 | *nr* | 0.98 | 0.98 | *nr* |
| Phillips et al. (2006) | n/a | PRELIS 2.51, LISREL 8.51 | 0.045 | 1397.999 | *nr* | 0.91 | *nr* | *nr* |
| Roberts et al. (1990) | n/a | LISREL V1 | *nr* | *nr* | *nr* | *nr* | *nr* | *nr* |
| Skriner and Chu (2014) | n/a | Mplus 6.11 | 0.43 | *nr* | 0.961 | 0.966 | *nr* | *nr* |
| Tatar et al. (2013) | n/a | PARSCALE 4.1, Amos 16.0 | 0.11 | 2223.37 | *nr* | *nr* | *nr* | *nr* |
| *nr = not reported; n/a = not applicable (CFA)* | | | | | | | | |
